# A review on control of droplet motion based on wettability modulation: principles, design strategies, recent progress, and applications

**DOI:** 10.1080/14686996.2022.2116293

**Published:** 2022-09-06

**Authors:** Mizuki Tenjimbayashi, Kengo Manabe

**Affiliations:** a International Center for Materials Nanoarchitectonics (MANA), National Institute for Materials Science (NIMS), Tsukuba, Ibaraki, Japan; b Research Institute for Advanced Electronics and Photonics, National Institute of Advanced Industrial Science and Technology (AIST), Tsukuba, Ibaraki, Japan

**Keywords:** Droplet manipulation, wetting, hydrophobic, functional interface, biomimetics, responsive materials, gradient structure, surface modification, superwetting, thin film

## Abstract

The transport of liquid droplets plays an essential role in various applications. Modulating the wettability of the material surface is crucial in transporting droplets without external energy, adhesion loss, or intense controllability requirements. Although several studies have investigated droplet manipulation, its design principles have not been categorized considering the mechanical perspective. This review categorizes liquid droplet transport strategies based on wettability modulation into those involving (i) application of driving force to a droplet on non-sticking surfaces, (ii) formation of gradient surface chemistry/structure, and (iii) formation of anisotropic surface chemistry/structure. Accordingly, reported biological and artificial examples, cutting-edge applications, and future perspectives are summarized.

## Graphical abstract

**Figure:**
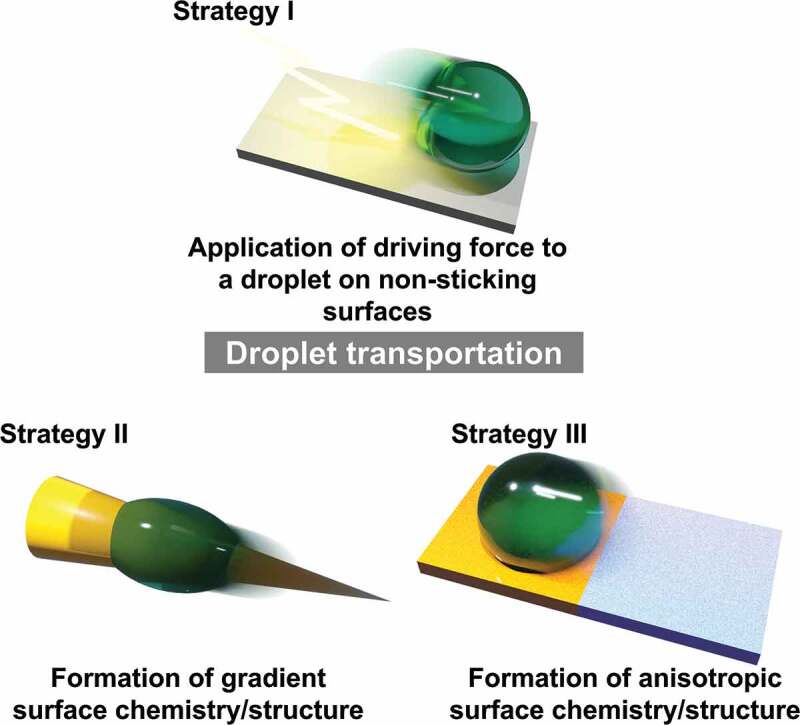

## 1. Introduction

Liquid transportation is vital in biology, industrial processes, and biomedical systems. The controllable manipulation of droplets is essential for various applications, including printing, fluidics, water harvesting, heat transfer, and energy generation. Droplets can be manipulated by applying driving forces to droplets while preventing undesirable adhesion to the contacting media. This requirement is realized by controlling the interaction and response of the surface and interfaces of the droplets, which significantly influence wetting dynamics [1,2].

**Figure 1.**
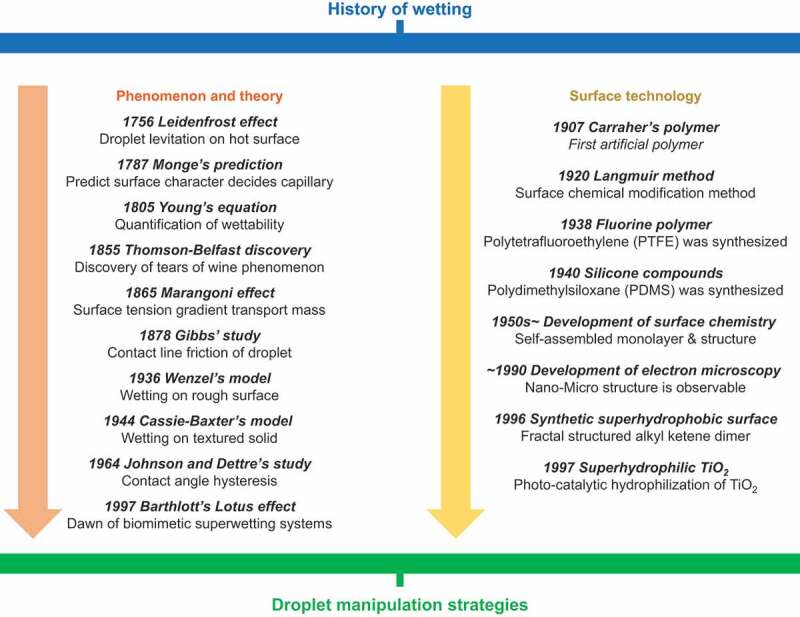
History of wetting from the perspective of fluid mechanics and materials science for droplet manipulation strategies.

Wetting dynamics control droplet behavior. Surfaces with controlled wettability have been extensively investigated since 2000 [3]. The wetting phenomenon was first treated academically over 200 years ago. Figure 1 summarizes the significant findings on the wetting phenomenon through the years.

First, we summarize the significant findings from the perspective of phenomenon and theory. In 1756, the Leidenfrost phenomenon was discovered in which droplets levitate on a superheated substrate [4]. In 1787, Monge reported that capillary action was an outcome of the superficial nature of the liquid [5]. Young presented a quantitative study of wetting phenomena in 1805 [6]. In his essay, he quantified the wettability of a droplet in terms of the contact angle. He showed that the contact angle is determined by the cohesive force of the droplet and the interfacial interaction (interfacial tension or capillary force) with the contacting substrate. The phenomenon wherein a gradient in the capillary force becomes the driving force for mass transfer between fluids is called the Marangoni effect. This effect was recognized by Thomson and Belfast in the ‘tears of wine’ phenomenon in 1855 [2] and studied by Marangoni in 1865 [7]. In 1878, Gibbs identified a frictional resistance in the movement of droplet contact lines [8]. Wenzel proposed models for droplet wetting behavior on rough surfaces in 1936 [9], followed by Cassie and Baxter in 1944 [10]. In 1964, Johnson and Dettre reported the relationship between droplet adhesion behavior (contact angle hysteresis) and surface roughness [11]. These studies laid the foundation for the classical theory of wetting. They suggested that surface chemical composition and structure are crucial in controlling wettability.

From the materials science perspective, in 1907, Ollivier reported water-repellent materials made of soot and lycopodium [12]. In the same year, Carraher synthesized polymers artificially for the first time [13]. Langmuir reported surface modification methods (around 1920) [14]. The fluorinated polymer polytetrafluoroethylene (PTFE) was synthesized in 1938, and the silicone compound polydimethylsiloxane (PDMS) was synthesized in 1940 [15]. Research in surface science and monolayers began to develop in the 1950s [16]. Around 1990, the development of microscopy technology enabled the analysis of nanoscale structures; consequently, several studies investigated materials to control wettability. In 1996, Onda et al. reported artificial superhydrophobic materials [17]. In 1997, photocatalyst-induced superhydrophilicity of titanium dioxide was reported [18]. These studies led to widespread research on surfaces not wetted by liquids [19]. Examples include liquid marble reported by Aussillous and Quéré in 2001 [20], superoleophobic surfaces reported in 2007 [21], and other liquid anti-adhesion surface technologies such as liquid-infused surface (LIS) reported in 2005 and after [22–25]. Several technologies have reported transporting droplets by applying a driving force on these non-sticking surfaces (Strategy I: Application of driving force to a droplet on non-sticking surfaces).

In 1997, Barthlott reported the superhydrophobic characteristic of the lotus leaf [19]. Since this discovery, biomimetics has been actively studied to emulate the wetting phenomena in nature using nano – microscale structural analysis [26]. Several cases of effective transport of water droplets in nature have been reported since 2000 [27–29]. A variety of droplet transport technologies that mimic or artificially reproduce these cases have also been reported. In nature, these phenomena have been observed in gradient structures and are effective for self-propelled droplet transport. Several artificial droplet transport techniques that effectively utilize these phenomena and the Marangoni effect have also been reported (Strategy II: Formation of gradient surface chemistry/structure).

Research on the frictional force of droplets has also matured, leading to modifying surface materials. In 1992, Abbott et al. reported wetting patterning using the micromachining process of the ‘biphilic’ monolayers [30]. Numerous studies have reported how patterning can limit droplet motion by imparting anisotropy to the droplet frictional force (Strategy III: Formation of anisotropic surface chemistry/structure).

Although various review papers have summarized the droplet manipulation studies [15,27–34], this review categorizes the strategies for transporting droplets into three types (Strategy I – III) and reports the design methods and features to realize them; furthermore, the actual examples concerning these strategies are discussed. This review shall serve as a guideline for the further development and application of droplet transport technology.

## 2. Design strategies

### 2.1. Overview of strategies

**Figure 2.**
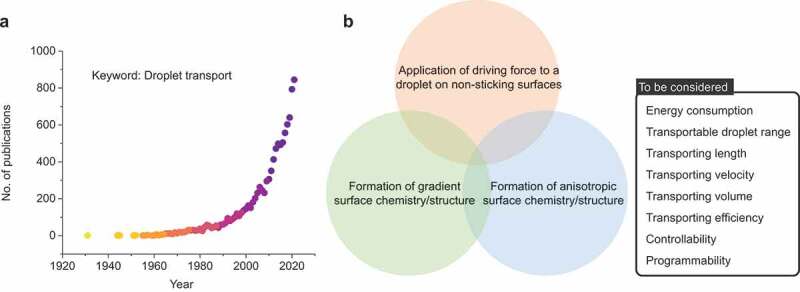
(a) Rapid increase in research interest (number of papers) on the topic of “droplet manipulation” by Scopus. (b) Strategies to transport droplets and parameters to be considered.

Figure 2(a) outlines the number of published studies on droplet transportation. The number of publications has increased dramatically since 1990. Reported strategies to transport droplets can be classified into the following three main strategies (Figure 2(b)):

Strategy I: Application of driving force to a droplet on non-sticking surfaces

Strategy II: Formation of gradient surface chemistry/structure

Strategy III: Formation of anisotropic surface chemistry/structure

The parameters involved are energy consumption for liquid transport, transport distance, transport speed, transport volume, transport efficiency (minimization of adhesion losses), and controllability/programmability. We elaborate on the principles and design strategies and summarize the previous works in the following section.

### 2.2. Strategy I: application of driving force to a droplet on non-sticking surfaces

#### 2.2.1. Mechanics

**Figure 3.**
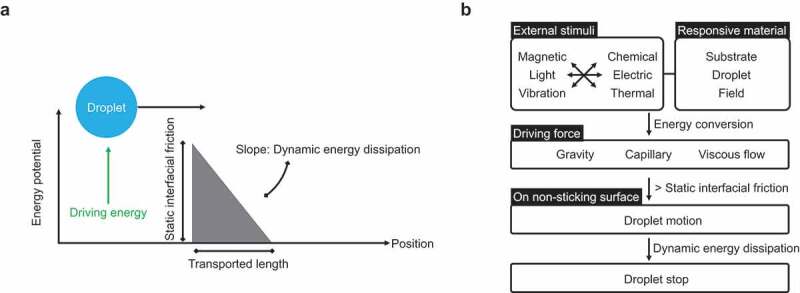
Droplet transportation strategies based on the application of driving force to a droplet on non-sticking surfaces. (a) Schematic of droplet energy. (b) Droplet transportation flow chart.

The first strategy involves applying a driving force to a droplet on a non-sticking surface. As shown in Figure 3(a), the droplet is placed statically on the non-sticking surface, and a driving force is applied. When the energy transferred to the droplet exceeds the energy barrier derived considering the frictional force of the droplet on the non-adhered surface, the droplet moves. The transport distance is determined by the energy transferred to the droplet and the energy dissipation rate during the motion. To provide a driving force to a droplet, an external stimulus (magnetic force, light, vibration, chemical reaction, electricity, or heat) and a responsive interfacial material (droplet, substrate, or field) are required (Figure 3(b)). These stimuli are converted into energy for driving the droplet mediated by the responsive interface, finally providing the droplet with the required driving forces in the form of gravity, capillary forces, and viscous flow. When these driving forces outweigh the frictional forces on the contacting surfaces, the droplet begins to move. The droplet motion stops when the energy is dissipated.

#### 2.2.2. Non-sticking surfaces

**Figure 4.**
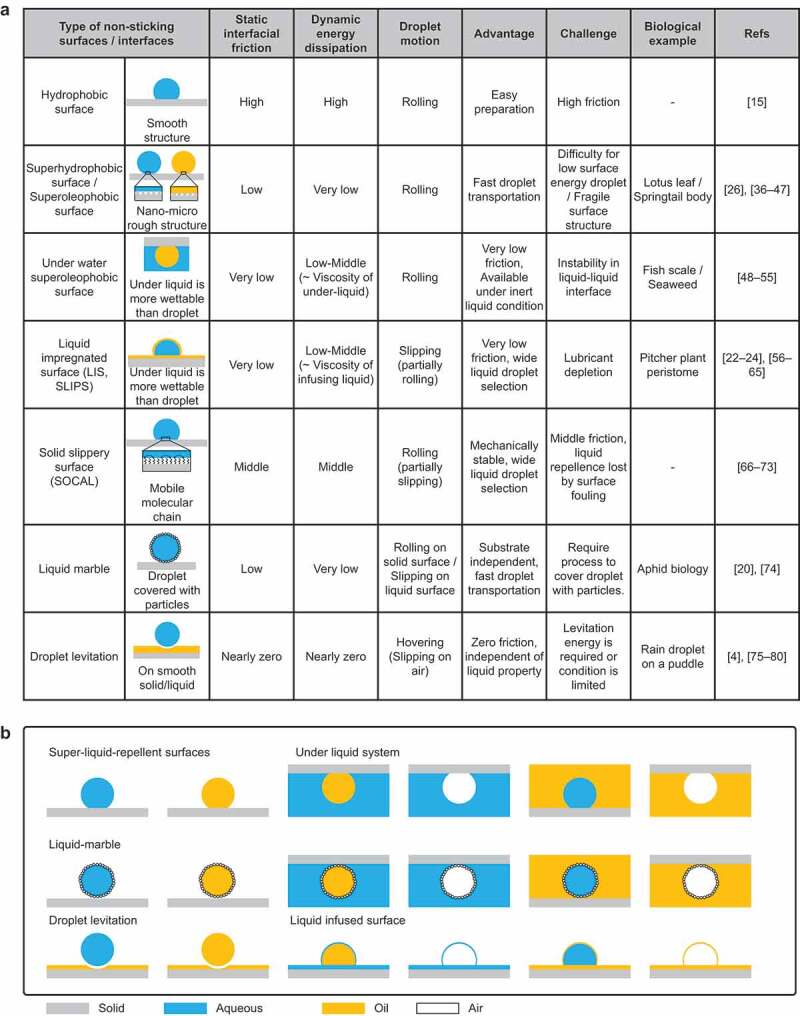
(a) Summary of non-sticking surfaces and their properties. (b) Variation in non-sticking surfaces/interfaces.

This strategy requires surfaces with low frictional resistances to the liquid. Figure 4(a) summarizes the typical low-friction interfaces and their characteristics. The origin of the frictional force of the droplet on hydrophobic surfaces has been discussed by Daniel et al [35].

Hydrophobic surface: Hydrophobic surfaces are formed by monolayers of water-repellent molecules or hydrophobic polymers (PDMS, PTFE) [15]. These surfaces have higher static and dynamic friction forces than the other non-sticking surfaces but can be easily developed.

Superhydrophobic and superoleophobic surfaces: These surfaces have nano- to micro-scale convex structures on hydrophobic surfaces [26]. In particular, generating a re-entrant structure and fluorinated or double re-entrant structure is required to develop a super oil-repellent surface [36–38]. Droplets cannot penetrate the micro-scale structure on these surfaces [39], resulting in a smaller contact area with the droplet; the droplets become spherical at contact angles >150° and rolls off with negligible tilting angle and contact angle hysteresis [40]. These droplets exhibit a static frictional force and low energy dissipation, enabling high-speed droplet transport [41]. In nature, lotus leaves are superhydrophobic [42], and springtail bodies are superoleophobic [43]. Challenges include the difficulty of adapting to low-surface-energy liquids such as organic solvents and fluorinated liquids [44] and the mechanical instability of the microstructure [45–47].

Underwater superoleophobic surfaces: Oil rolls off the surface with a spherical shape in water [48–50]. These surfaces are composed of hydrophilic polymers or hydrogels with a water-film suppressing direct contact with oil droplets [51,52]. Therefore, the static frictional force is infinitesimally small. Energy dissipation depends on the viscosity of water. This concept can be extended to superaerophobicity in water or oil and superhydrophobicity in oil [53,54]. The advantage of this method is the possibility of droplet transport technology in inert liquids. The droplets and surrounding liquid must be immiscible. However, the droplet – surrounding liquid interface is, in some cases, unstable. In nature, fish scales [48] and seaweeds [55] are superhydrophobic in water.

Liquid-infused surface(s)/slippery liquid-infused porous surface(s) (LIS(s)/SLIPS(s)): An LIS is formed by infusing the lubricant into a lubricant-philic porous structure or using desirable intermolecular interaction to retain the lubricant, allowing the immiscible liquid to slide down with a negligible tilting angle [22–24,56]. Therefore, the frictional force at the interface is minimal but depends on the lubricant viscosity for energy dissipation [57,58]. However, the sliding droplets deplete the lubricant [59,60]. Depending on the choice of the lubricant, the motion of droplets of simple liquids such as water, oil, or organic solvent [24,61] as well as complex liquids such as blood [62,63] or other complex substances [64] can be facilitated. In addition, LISs have a self-healing property [65]. Even if the porous structure is damaged, the lubricant can immediately wet the surface and spread out, restoring the original lubricating ability.

Solid slippery surfaces (liquid-like surfaces, slippery omniphobic covalently attached liquid-like (SOCAL) surfaces) [66,67]: These surfaces have been popular since they were first intensively studied by Fadeev et al [68,69]. Contact angle hysteresis is increased by chemical or structural defects, which prevents most surfaces from improving their slip-off performance [70]. SOCAL [66], a solid slippery surface, is a smooth surface with hydrophobic chain molecules covalently bonded to the surface. It is characterized by extremely low contact angle hysteresis, allowing droplets to slide off. In contrast with other surfaces, the sliding angle decreases as the surface tension of the liquid to be slid decreases [71]. Its fabrication method is simple, and it can demonstrate durability in extreme environments such as high temperatures and pressures [72]. However, the smoothness of the surface hinders increasing the contact angle, and the contact area between the surface and the droplet increases, resulting in a slower sliding speed [73].

Liquid marble: This is a droplet covered with hydrophobic powder [20,74]. The contacting surface is no longer wetted by the droplet, and the droplet can be handled as an elastic solid. The droplet can roll off the solid surfaces independent of its wettability and float on the liquid pool. This is because the hydrophobic powder prevents direct contact between the contacting surface and the droplet surface.

Droplet levitation: This occurs under specific conditions, wherein drops impacting at low Weber numbers levitate on solid surfaces. Droplets can also levitate on scorching surfaces; this is known as the Leidenfrost phenomenon [4]. These techniques are independent of the surface energy of the droplet and enable it to move with zero friction [75]. However, the energy required to levitate the droplet and the levitation period are limited [76–80].

These techniques can also be realized by replacing the droplet and/or surrounding fluid with other fluids such as water, oil, or air (Figure 4(b)). The variation shown in the figure below has been reported.

#### 2.2.3. Which force dominates droplet mechanics?

**Figure 5.**
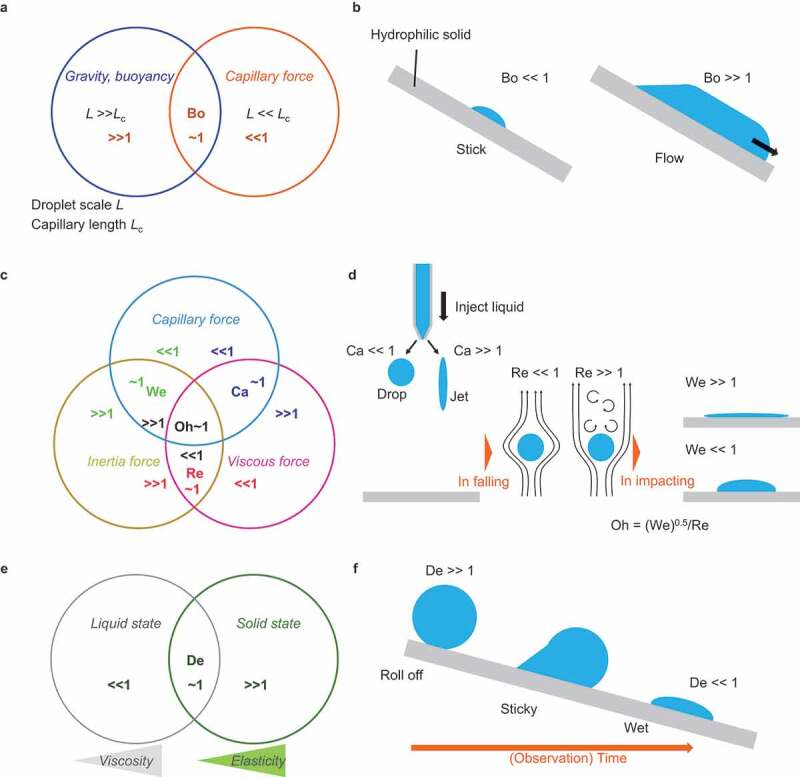
Consideration of dimensionless parameters for the determination of the force that dominates droplet mechanics.

When considering droplet transport, the relative dominance of various forces acting on the droplet should be considered. Figure 5 summarizes the dimensionless parameters that can be used as a guideline.

First, consider a droplet at rest. Figure 5(a) considers whether capillary force or gravity (buoyancy) is dominant. Bond number expresses the balance between capillary force and gravity as Bo = [Gravity or Buoyancy]/[Capillary force] = *ρgL*^2^/*γ*, where *ρ* is the liquid density, *g* is the gravitational acceleration constant, *L* is the characteristic length, and *γ* is the liquid surface tension. Bo < <1 implies that the liquid surface tension dominates the mechanics rather than gravity and *vice versa*. The characteristic length for Bo = 1 is called capillary length *L*_c_ = [*γ*/(*ρg*)]^0.5^ (typically 2–3 mm), which gives Bo = [*L*/*L*_c_]^2^ [78].

Figure 5(b) considers the droplets cast on a hydrophilic surface. When the droplet radius is L = 0.1 mm, significantly smaller than Lc (that is, Bo   10 − 3), the droplet wets and rests on the tilted hydrophilic surface with a spherical cap shape. When the droplet radius is L = 100 mm, significantly greater than Lc (that is, Bo   103), the droplet attains a puddle shape and flows on the hydrophilic surface.

Next, we consider droplet transportation. The balance between capillary, viscous, and inertia forces influences the droplet motion in the dynamic condition. Figure 5(c) summarizes the dimensional numbers. Capillary number expresses the balance between the capillary and viscous forces and is given by Ca = [Viscous force]/[Capillary force] = *ηV*/*γ*, where *η* is the viscosity and *V* is the characteristic velocity. Reynolds number Re = [Inertia force]/[Viscous force] = *ρLV*/*η* balances the inertia and viscous forces. Weber number We = [Inertia force]/[Capillary force] = *ρLV*^2^/*γ* balances the inertia and capillary forces.

Figure 5(d) considers the printing process: a liquid droplet is ejected on a hydrophilic surface. When a droplet is ejected, it is subjected to viscous resistance that depends on the ejection speed [81]. For example, when low-viscosity liquid water is gently ejected at 1 mm/s (that is, Ca   10^−5^), the spherical droplets fall as the capillary force dominates the phenomenon. However, when a high-viscosity liquid, glutinous starch syrup (*η* 10^6^ mPa s), is ejected at 1 m/s (that is, Ca   10^4^), droplets do not form, and the liquid is ejected under laminar flow. When the ejected droplet falls, the capillary force applied to the droplet is constant, and the droplet is affected by the inertial force due to the fall and the viscous resistance of the air. The airflow state depends on the air velocity (that is, relative droplet velocity) and viscosity [41,82]. When Re<<1, that is, when the droplet size and falling velocity are small, or when the droplet falls into a viscous oil pool, laminar flow results, and the droplet falls straight down. However, when the droplet size and velocity are large (Re > >1), turbulent flow results behind the moving droplet, and the droplet falling behavior becomes unstable. Consider a droplet impacting the substrate [83]. When the impact velocity and droplet size are sufficiently large (that is, We>>1), the droplet is deformed immediately after impact due to the impact pressure (water hammer pressure and dynamic pressure), regardless of the wettability of the droplet. Moreover, when the droplet is gently dropped (that is, We<<1), the droplet is not significantly deformed upon impact, and the shape is determined by the wettability. Ohnesorge number is expressed as Oh = [Inertia force]^0.5^× [Capillary force]^0.5^/[Viscous force] = We^0.5^/Re, indicating the relative dominance of viscous force over the other two forces. These numbers indicate the dynamic factors in the droplet motion and are influenced by the physical properties of the fluid and the experimental system [84,85].

When dealing with high-viscosity liquids [86,87], such as polymer melts, it is necessary to consider the effect of the relaxation time of the liquid. Deborah number De = [Observation time]/[Relaxation time] considers the treatment of the high viscosity liquid (Figure 5(e)). For example, if the viscosity of a liquid is extremely high, such as that of glutinous starch syrup or peanut butter, the time it takes to deform due to capillary force is extremely long, and it behaves similar to a viscoelastic body for a short time. Such a liquid can be treated as a solid (elastic body) when considering periods shorter than the deformation time and as a liquid (viscous) on a time scale sufficiently longer than the relaxation time (that is, De>>1). Figure 5(f) shows a viscous liquid drop (ideally in droplet form) on a hydrophilic substrate. For a short period (that is, De>>1) from the moment the droplet contacts the substrate, it exhibits solid-like behavior and rolls off. However, as the observation time increases, it gradually begins to exhibit liquid-like behavior, becomes sticky, and finally wets the substrate (that is, De<<1). When handling highly viscous liquids, the decision to treat the droplets as solid-like or liquid-like is important.

#### 2.2.4. Summary of strategies

**Figure 6.**
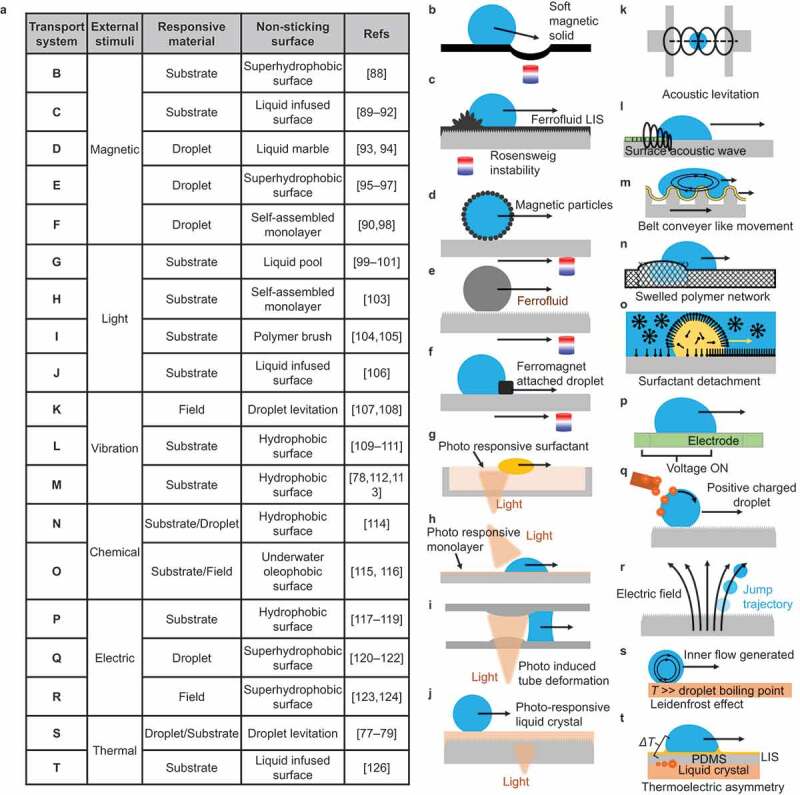
Natural and artificial droplet transport based on the application of driving force to a droplet on non-sticking surfaces.

Examples of droplet transportation based on Strategy I are shown in Figure 6. In all cases, the external stimulus (magnetic force, light, vibration, chemical reaction, electricity, or heat), responding material (substrate, droplet, surrounding environment, or a combination thereof), and low-adhesion surface are combined to achieve droplet transport.

In Figure 6(b–f), a magnetic force is converted to droplet driving force. Strategies such as deforming a magnetic substrate by rendering it superhydrophobic and moving droplets by gravity (Figure 6(b)) [88], using magnetic fluid in LIS to deform the film by Rosensweig instability spiking phenomenon (Figure 6(c)) [89–92], liquid marbling with magnetic particles (Figure 6(d)) [93,94], manipulating magnetic water solutions on a superhydrophobic surface (Figure 6(e)) [95–97], and transporting magnetic hydrophilic beads by attaching magnetic hydrophilic beads to droplets (Figure 6(f)) [90,98] have been reported. The advantage of magnetic transport is that the droplet position can be adjusted in situ depending on the position of the magnet. Another advantage of using permanent magnets is that no external energy is required other than the energy used in changing the position of the magnet.

In Figure 6(g–j), light-induced droplet transport is reported. The cis – trans isomerization of azobenzene causes a change in the interfacial tension of the light-exposed spot that in turn induces a capillary force on the droplet, resulting in its transport. In Figure 6(g) [99], a photo-responsive surfactant is adsorbed on a liquid pool, and immiscible droplets are transported. Photo-induced chemical change or depletion of the surfactant varies the surface tension, and capillary force drives droplets in the pool [100,101]. Another strategy uses the photothermal effect to drive droplets as the surface tension varies with temperature [102]. Figure 6(h) shows droplet transport on a surface employing a photo-responsive single molecule [103]. Figure 6(i) shows droplet transport by capillary force inside a light-swollen tube [104,105]. Figure 6(j) shows droplet transport using a light-responsive liquid crystal as a lubricant in LIS [106]. The advantage of light-induced droplet transport is that it can be remotely controlled over long distances and through walls using transmitted lasers. In addition, droplet motion can be programmed in-situ.

Figure 6(k–i) show droplet transport by ultrasonic waves, and Figure 6(m) shows droplet transport by periodic vibrations. Figure 6(k) shows droplet transport by ultrasonic levitation that is contactless [107,108]. Figure 6(i) shows droplet transport by surface acoustic waves [109–111]. In Figure 6(m), droplets are transported similar to a conveyor belt transport [78,112,113].

Figure 6(n–o) show the periodic motion of droplets driven by molecular diffusion. Figure 6(n) utilizes the swelling of a water-repellent polymer [114]. The swelling is due to the diffusion of the transporting droplet into the polymer matrix. Figure 6(o) shows the transport of oil droplets in water [115]. Oil droplet movement is caused by desorption and reabsorption of surfactant to the interface. This type of droplet movement is observed for a water droplet in an oil pool [116].

Figure 6(p–r) show droplet transport by electricity. Since polar droplets change their shape due to surface charging, electrically adjustable wetting has been widely studied as electrowetting. Figure 6(p) shows an example of droplet transport by electrowetting [117–119]; an induced effect between an electrode and a droplet on a solid surface attracts the droplet to a charged site. In Figure 6(q), droplets are transported using electrostatic repulsion on a superhydrophobic surface [120–122]. Figure 6(r) shows the control of droplet jump trajectory along an electric field on a superhydrophobic surface [123,124]. In controlling the charged droplet, both the coating surface and substrate permittivity also influence the droplet sliding behavior [125].

Figure 6(s–t) show droplet transport owing to thermal stimuli. Figure 6(s) shows a droplet being driven by droplet volatilization flow, the so-called Leidenfrost effect [77–79]. Water droplets volatilize rapidly on a hot solid substrate (>250 °C), forming a vapor film between the substrate and the droplets. The vapor film acts as an adiabatic film; therefore, the droplet floats on the surface and moves without boiling. The control method of Leidenfrost is explained in Strategy III below. Figure 6(t) is a droplet transport system that converts temperature into voltage through the interaction of a thermoplastic polymer and piezoelectric crystals [126]. In this manner, some methods convert external stimuli into energy using the physical properties of the material. The key is to reduce energy dissipation at the droplet transport interface while providing a driving force to the droplet.

### 2.3. Strategy II: formation of gradient surface chemistry/structure

#### 2.3.1. Mechanics

**Figure 7.**
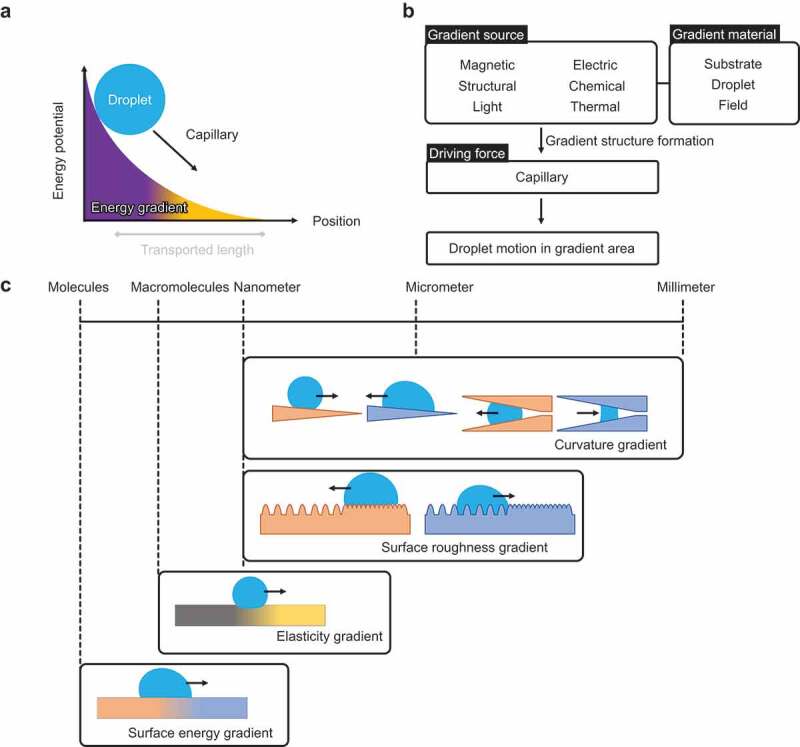
Droplet transportation strategies based on gradient surface structure/chemistry.

Strategy II is based on gradient surface chemistry and/or structure. By applying an energy gradient to the droplet contact interface, the droplet spontaneously moves in the direction of lower energy (Figure 7(a)). The slope of the gradient determines the velocity of the droplet, and the length of the gradient determines the transporting distance. In contrast with Strategy I, in Strategy II, typically, the preset energy gradient spontaneously moves the droplet a fixed distance. Moreover, non-sticking surfaces are not used.

Figure 7(b) explains the droplet transportation. The sources that create gradients are magnetic, electrical, structural, chemical composition, light, and heat, which are diffusive stimuli and have a spatial gradient intensity. The substrate, droplet, or field responds to the stimuli, forming the gradient structure and surface energy. As a result, the droplet is driven by capillary forces along the gradient direction.

Figure 7(c) summarizes the gradient surfaces with different length scales. The length scales of the gradient structure extend from the millimeter scale to the molecular scale. Curvature gradient is formed in the nanometer to millimeter scale. When a droplet contacts a conical solid, the droplet shape becomes anisotropic. Because the three-phase contact lines by the tip of the needle – droplet–air and that by the root of the needle – droplet–air have different lengths, the capillary forces are not balanced in the direction of the needle root and the needle tip, and the droplet moves spontaneously. The droplet transport direction depends on the wettability. Spontaneous droplet transport is also observed in a tube with a gradually narrowing mouth. Surface roughness gradient is formed in the nanometer to sub-millimeter scale. According to Wenzel theory, droplet wettability is influenced by surface roughness: a hydrophobic surface becomes more hydrophobic due to surface roughness and vice versa. The wettability of a surface with the same physical properties changes when its roughness varies. When a droplet is placed on a surface with a roughness gradient, it spontaneously moves in the direction of higher wettability. An elastic gradient is formed by the macromolecular scale design, such as the cross-linking degree of polymer networks. When the droplet is on a soft solid whose Young’s modulus is less than or equal to the order of the interfacial tension, the tension deforms the interface, and the direction in which the surface tension acts changes. When there is a gradient in elastic modulus, the degree of deformation of the interface by the droplet changes along with the horizontal components of the interfacial tension between the flexible side and the hard side; therefore, capillary force becomes anisotropic and drives the droplet. Surface energy gradient, including electron and spin, is the molecular-scale design. A gradient in surface tension causes the spontaneous transport of droplets in the direction of a more wettable area. This wettability is determined by the surface chemical composition and electrical/magnetic interactions.

#### 2.3.2. Summary of strategies

**Figure 8.**
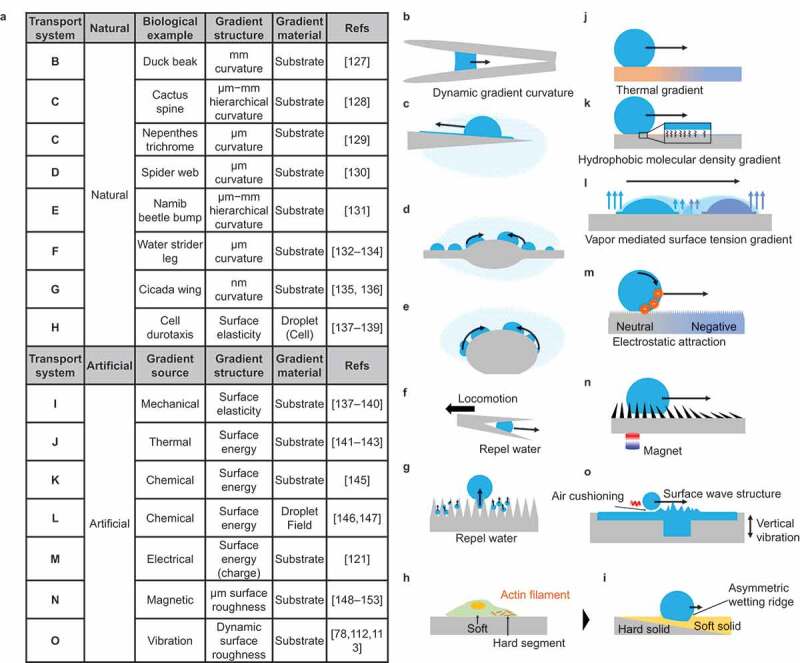
Natural and artificial droplet transport based on gradient surface structure/chemistry.

Examples of droplet transportation based on Strategy II are shown in Figure 8(a). This type of droplet transportation is often found in nature. The material and process approach to forming gradient structures using technology has been reported. Figure 8(b–h) show the spontaneous transport of water droplets in nature using a curvature gradient structure. As shown in Figure 8(b), a waterfowl needs to transport water droplets from the tip of its beak to its root to drink water; a gradient structure of curvature is used for this purpose [127]. As shown in Figure 8(c), cactus spines and trichrome of nepenthes pitcher plant have a gradient curvature structure for the collection of water toward the cactus root [128,129]. The hair-like projections of the cactus use a similar mechanism to achieve the spontaneous transport of water droplets [128]. Figure 8(d) shows the phenomenon of water droplet collection in a spider web [130]. Spider webs have a micrometer-sized spinning structure, and water droplets are collected in the spinning structure. Figure 8(e) presents the water-harvesting mechanism of the Namib beetle [131]. The Namib beetle has a hump-shaped hierarchical projection on its back, and this structure enables water collection. In Figure 8(f), the legs of the water strider have curvature gradients to repel water [132–134]. Micro-scale curvature gradients are used to prevent the water from penetrating between the legs. In Figure 8(g), nanometer-scale curvature gradient structures are observed on the surface of cicada wings [135]. Experimental studies have reported that this structure effectively promotes microdroplet fusion and ultimately removes water from the wings [136]. Figure 8(h) shows Durotaxis, a form of cell migration [137–139]. Cells move by forming a stiffness gradient during their migration. Recently, studies that consider cells as droplets have been reported, and this is also included as an example of spontaneous transport by a gradient [140].

Figure 8(i–o) present the spontaneous transport of droplets using materials (artificial). In Figure 8(i), spontaneous transport of water droplets is achieved by forming a stiffness gradient on the solid surface [137–139]. The gradient change in thickness of the soft solid coating on a rigid substrate realizes the stiffness gradient. Droplet moves to the softer side. In Figure 8(j–m), droplets are transported by artificially forming a surface tension gradient (molecular scale effect). In Figure 8(j), the temperature dependence of surface tension is used to form a gradient of surface tension by thermal diffusion [141–143]. The maximum substrate temperature is typically less than the liquid boiling point to form a surface tension gradient thermally. When the substrate temperature achieves Leidenfrost temperature, a thermal air flow gradient is formed to transport volatile droplets [144]. In Figure 8(k), a surface tension gradient is formed by setting a gradient in the density of modified molecules [145].

In Figure 8(l), two drops of a liquid mixture of water and ethylene glycol are placed side by side to form a vapor density gradient with the vapor density between the two liquids as the maximum value [146,147]. Thus, the surface tension between the two liquids is larger than that outside because the volatility of water and the mixing ratio of ethylene glycol decreases with the vapor density. Consequently, an interfacial tension gradient is formed. In Figure 8(m), droplet transport is achieved by generating a charge gradient on a superhydrophobic surface [121]. The surface is modified with fluorinated hydrocarbons, and the surface is negatively charged by frictional charging when contacted by water droplets. The gradient is formed by controlling this charging ratio with the droplet contact area. In Figure 8(n), a gradient of surface roughness is formed [148–153]. By forming a needle-like structure of soft PDMS containing magnetic particles and using a magnet, a gradient of the needle-like structure is created by orienting the needle to the gradient magnetic field gradient, resulting in the formation of gradient surface roughness. In Figure 8(o), a surface roughness gradient is formed by using vibration [78,112,113]. Ripples are formed by vibrating the water surface, and these ripples decay, resulting in a surface roughness gradient that gradually flattens with the center of vibration as the roughest surface. In this study, the position of the ripples is controlled by the shape of the tank. When the tank vibrates vertically in a partially deep portion, that portion becomes the vibration source, and the droplets move toward the vibration source.

### 2.4. Strategy III: formation of anisotropic surface chemistry/structure

#### 2.4.1. Mechanics

**Figure 9.**
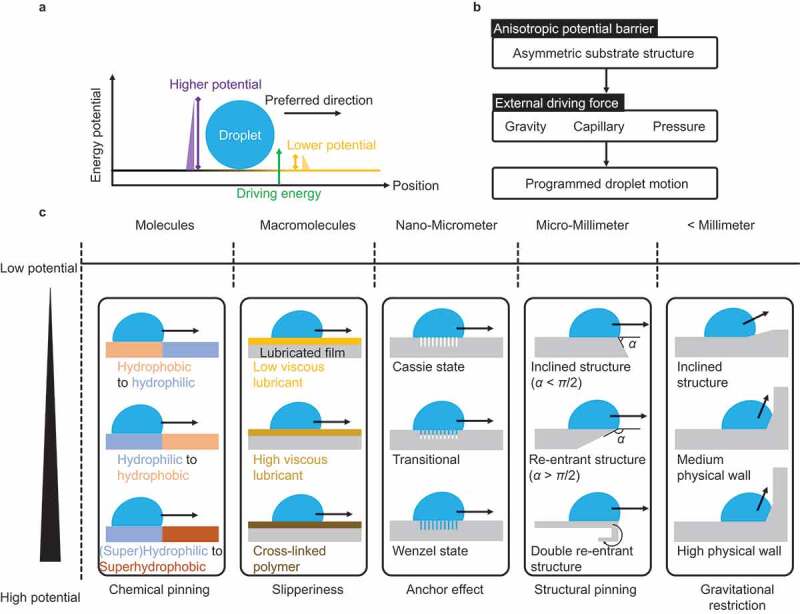
Droplet transportation strategies based on anisotropic surface structure/chemistry.

Strategy III is based on anisotropic surfaces (Figure 9). In this strategy, as shown in Figure 9(a,b), the energy potential is set anisotropically using a substrate with asymmetric surface chemistry and structure; the droplet moves in the direction of the lower potential when a force (for example, gravity, capillary, pressure) is applied to the entire field. Programmable droplet transport becomes possible. This potential is the barrier to the movement of the three-phase contact line, including the droplet. The surface design of the asymmetric structure is covered from molecular- to millimeter-scale, as summarized in Figure 9(c). The surface design in different length scales is explained below.

At millimeter-scale and above, the potential energy (gravitational restriction) is controlled by the physical wall based on macroscopic structure.

At the micrometer- to millimeter-scale, the pinning effect limits the droplet motion. When a droplet with contact angle *θ* moves over an edge of a solid surface with an inclining angle *α*, the droplet cannot move over until the angle exceeds *θ* + *α*. Thus, the energy potential for droplet motion increases with *α*. The so-called re-entrant structure with *α* > π/2 requires high energy to advance the droplet, which can prevent the liquid from spreading. Thus, the structure is used for the design of liquid-repellent surfaces. The double re-entrant structured surface exhibits super-repellent property, even to highly wettable liquids [44].

From the nanometer- to micrometer-scale, the pinning of the droplet between the rough structures (anchor effect) of the substrate limits the droplet motion. The potential energy increases with the degree of liquid invasion of the rough structure. When the droplet on the surface is in the Cassie state, the droplet does not invade the rough surface, the droplet substrate contact area is small, the droplet adhesion is weak, and the droplet slides off the slightly tilted surface. When the droplet/surface are in the Wenzel state, the droplet thoroughly wets the rough surface and firmly sticks to the surface. The interfacial state depends on the roughness feature and wettability [154]. Moreover, the Cassie-state droplet can transition to the Wenzel state by the application of external stimuli to the droplet, such as heating, lowering surface energy, or applying pressure [155].

Surface viscoelasticity plays an essential role in droplet motion on the macromolecular scale. When the surface is lubricated by low-viscous liquids immiscible with the targeting droplet (for example, silicone oil, fluorinated oil, or fatty acids as the lubricant and water as the droplet), the droplet slides off the surface with negligible contact angle hysteresis owing to the surface mobility. However, if the surface lubricant is considerably viscous or cross-linked, the surface mobility is lost, and the droplet requires high energy to slide off. Thus, the energy potential required to move droplets increases with the increase in the viscosity of the lubricant and solidification [156].

At the molecular scale, the pinning effect by the surface energy heterogeneity limits the droplet motion. When a droplet with contact angle *θ*_A_ moves over a surface with the contact angle *θ*_B_, the droplet cannot move over until the angle exceeds *θ*_B_. Thus, the energy potential for the droplet motion increases with *Δθ* = *θ*_B_ − *θ*_A_. When the droplet on a hydrophobic substrate area moves over a hydrophilic surface area, the droplet spontaneously moves to the hydrophilic area. This is because the energy potential for the hydrophilic area is smaller than that for the hydrophobic area (*Δθ* <0). However, when the droplet on the hydrophilic area moves over the hydrophobic area (*Δθ* >0), the droplet requires higher driving energy than the energy potential.

#### 2.4.2. Summary of strategies

**Figure 10.**
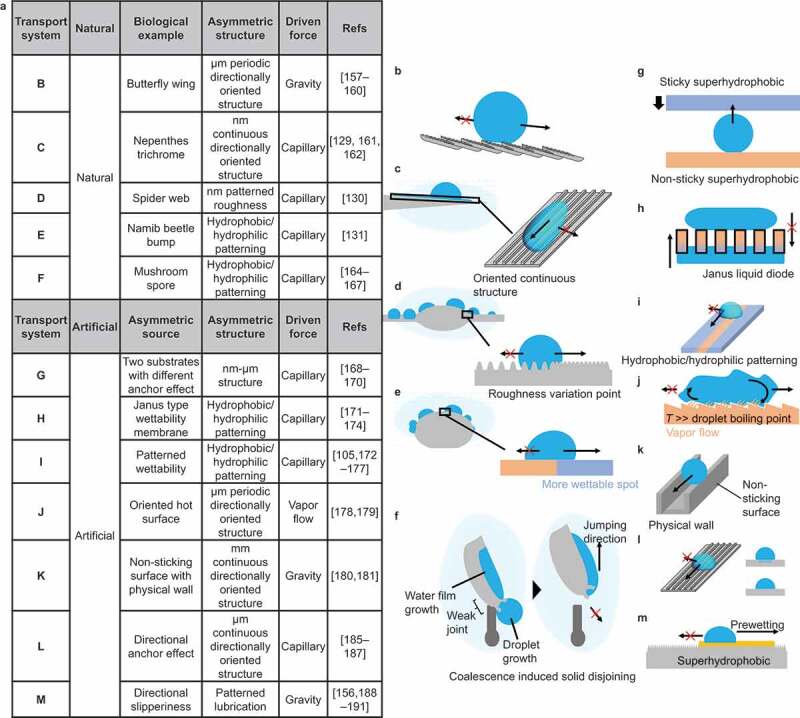
Natural and artificial droplet transport based on anisotropic surface structure/chemistry.

Examples of droplet transportation based on Strategy III are summarized in Figure 10(a). Figure 10(b–f) show the anisotropic surface structures in nature. The butterfly wing has anisotropic periodic surface structures on the micrometer scale (Figure 10(b)) [157–160]. Due to this anisotropy, the droplet sliding angle differs from the sliding direction. Figure 10(c) shows the structure of the trichrome of the nepenthes pitcher plant. In addition to the micrometer-scale curvature gradient structure (Figure 8(c)), the surface has a nanometer-scale groove structure [129,161,162]. This anisotropic structure limits the droplet movement to the parallel direction of the groove and directs the water collection. Figure 10(d) shows a spider web structure [130,163]. The spider web has a curvature gradient structure with knots, but the boundary between the knot and hair bundle parts has a different roughness feature, limiting the droplet transport direction.

Figure 10(e) shows the droplet harvesting technique of the Namib beetle [131]. In addition to the curvature gradient structure, the Namib beetle has a more hydrophilic top-of-the-knob-like projection, allowing collected water to remain at the top. Figure 10(f) shows the phenomenon of mushroom spores moving upward due to droplet coalescence [164–167]. The spores attach a water droplet to the hydrophilic site, and another contacting droplet is transported to this hydrophilic site. The surface tension change caused by droplet adhesion is converted into mechanical energy. Due to the weak joint of the spores, mechanical energy is used for moving upward. Figure 10(g–m) show a droplet transport technique using artificial anisotropic surfaces.

Figure 10(g) shows the transport of water droplets between superhydrophobic surfaces with different adhesion forces [168–170]. Even though the static contact angle is at a level of superhydrophobicity, the adhesion force varies with surface roughness feature. It is possible to move water droplets to a superhydrophobic surface with higher adhesion. Figure 10(h) shows directional liquid transportation using Janus porous membrane [171–174]. Janus materials have a hydrophobic side and a hydrophilic side. Water droplets on the hydrophobic side do not penetrate the pore on this membrane, but those on the hydrophilic side can soak into and penetrate the pore. Figure 10(i) shows a surface patterned with water repellent hydrophobic and water sticky hydrophilic surface stripes [105,175–177]. Due to chemical pinning, water droplets can move along the hydrophilic surface but do not move over the hydrophobic side. Figure 10(j) utilizes a periodic anisotropic ratchet structure to control the transport direction of water droplets in the Leidenfrost phenomenon [178,179]. The ratchet structure creates anisotropy in the vapor flow of the droplet and controls the direction of droplet movement. In Figure 10(k), a physical wall is formed to restrict the movement of water droplets on the superhydrophobic surface [180,181]. Several techniques for shaping superhydrophobic structures have been reported, including 3D printing [170,182], sculping [183], and direct coating to the shaped substrate [180,184]. The controlled physical wall allows programmable droplet movement. Figure 10(i) shows a micro-scale groove structure [185–187]. This anisotropic structure limits droplet motion to the parallel direction of the grooves and directs water collection. A similar structure at the micrometer scale is observed on the rice-leaf surface. Figure 10(m) shows an oleophilic surface partially impregnated with lubricant to form a patterning of rigid and fluid areas [156,188–191]. On this surface, the movement of water droplets is limited to the lubricant-impregnated areas, which can restrict their movement.

#### 2.4.3. Directional continuous liquid transport

**Figure 11.**
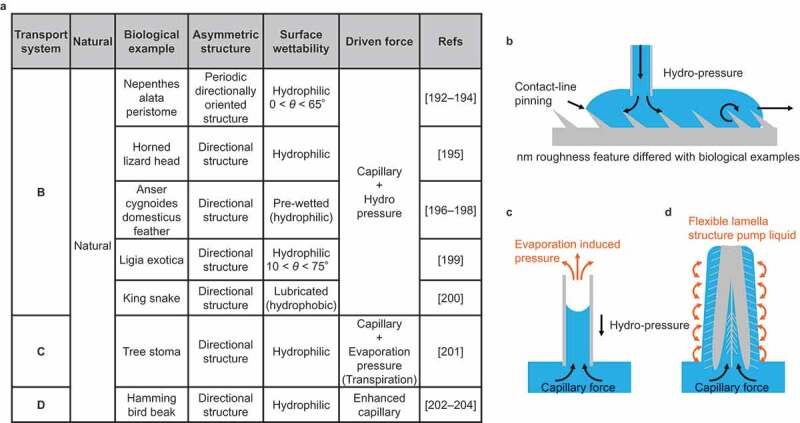
Natural directional continuous liquid transport.

Although not strictly ‘droplet’ transport, this section introduces a technique limiting the transport direction of a continuous liquid to one direction (Figure 11). Several such surfaces in nature have been reported, as summarized in Figure 11(a). The bioinspired surface structures mimicking the natural mechanisms have been reported. Figure 11(b) shows that natural surfaces exhibit unidirectional water spreading. These surfaces have a periodic directionally oriented structure seen in the nepenthes’ peristome [192–194], head of the horned lizard [195], feather of the Anser cygnoides domesticus [10,36,196–198], leaf of the Ligia exotica [199], and the scale of the king snake [200]. These surface structures and the critical wettability of the spreading liquid are strictly different; however, the main driving forces of the liquid are capillary and hydro-pressure. Several biomimetic surfaces mimicking this structure have been reported in the 2010s [33]. Figure 11(c) shows the water spreading from the root to the leaf of the woods and plants called transpiration [201]. Wood has porous microchannels inside, parallel to the stem, for water transportation. The driving force is not composed of just the capillary force; it is assisted by the pumping force resulting from the change in water evaporation pressure. Figure 11(d) illustrates the water drinking process through the beak of the hummingbird [202–204]. Water transport via hummingbird beak is based on a combination of beak macrostructure (Figure 8(b)) and microscale flexible lamella structure. The lamella structure pumps the water.

## 3. Potential applications

**Figure 12.**
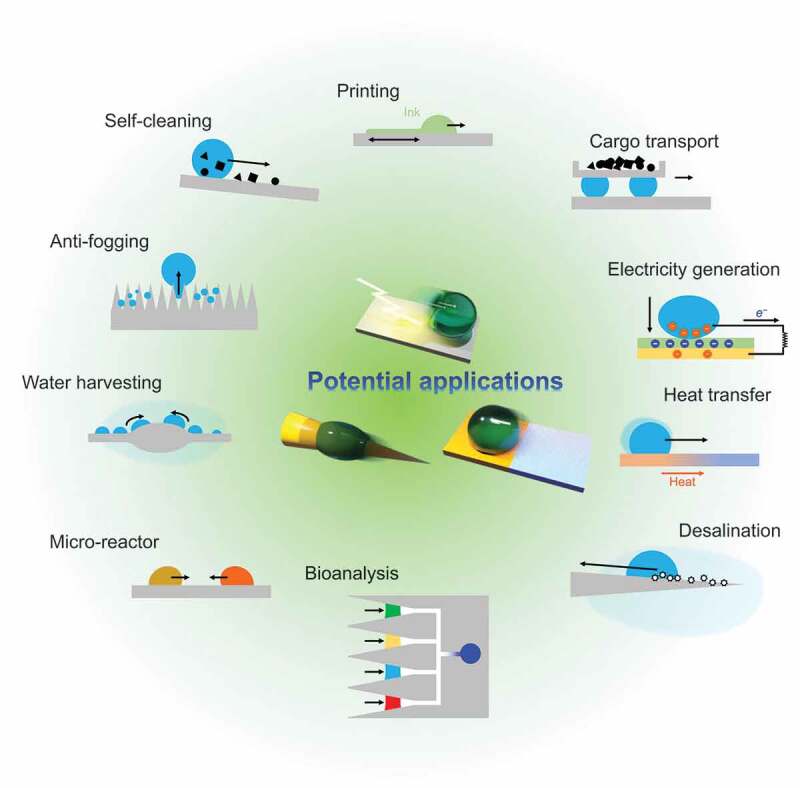
Applications of droplet transportation.

Finally, we summarize the potential applications of droplet transportation (Figure 12) and discuss the challenges limiting the practical use and perspectives.

### 3.1. Printing

Inkjet printing has long been used as a technique for accurate patterning of droplets. In a typical inkjet printing method, droplets are extruded by a piezoelectric (piezoelectric) element, but this approach is difficult to apply in the case of highly viscous Newtonian or yield-stress fluids. Foresti et al. reported a new method called acoustophoretic printing for precisely positioning various fluids and soft materials [205]. Subwavelength Fabry – Perot resonators were used to generate precisely localized acoustic phoresis forces two orders of magnitude greater than gravity to precisely control the dispensing of microliter to nanoliter volume droplets.

Furthermore, there is a technique for precise patterning by controlling the wetting gradient on the substrate side. Li et al. applied a hydrophobic/hydrophilic patterning treatment to a printed circuit board and applied a metal ink solution to the entire substrate [206]. The ink was transported to the line-shaped hydrophilic areas formed by patterning. After drying, fine printing with a minimum line width of 600 nm was achieved. Popova et al. fabricated superhydrophilic spots arranged at regular intervals on the superhydrophobic surface [207]. Droplet arrays were fabricated by tracing the substrate with water or an aqueous solution, causing the droplets to adhere to the substrate in a lattice.

### 3.2. Micro-reactor

Conventional microreactors form microchannels in tubules or on surfaces, through which fluids flow to synthesize specific substances at the junctions of the microchannels. Although these are industrially established systems, surfactants that compromise product purity, elimination of contamination, and customization along with the fluid and synthesized substance remain challenging.

Moreover, the realization of controlled water droplet transport described in this review allows microdroplets as reaction spaces for the synthesis of specific substances. This is also known as an open droplet microfluidic channel that can address the challenges of conventional microreactors described above. Surfaces that allow individual droplets to be manipulated individually and locally are particularly suitable.

For example, Xu et al. designed an open microfluidic platform using thermotropic liquid crystals [208]. They found that by stabilizing a porous liquid crystalline polymer network and a thermotropic liquid crystal meso-source and controlling the orientation order of its liquid crystal molecules, the stick/slip of water droplets on the surface could be manipulated.

### 3.3. Cargo transport

Surface charge density gradient-mediated droplet transport proposed by Sun et al. has achieved high-speed and long-distance droplet transport on a superhydrophobic surface [121]. It was shown that four water droplets could be used as a wheel on the surface, and solid cargo could be carried on the droplets.

Liquid crystal-based transport surfaces can move tiny droplets of water when the orientation order of the liquid crystal molecules changes when light is input as energy. Xu et al. exploited this phase transition property of liquid crystal molecules to recover materials placed on the surface [208]. They encapsulated counter-soluble dyes such as ethyl orange, rhodamine B, methyl orange, and methylene blue in reverse emulsions. They demonstrated that UV irradiation could induce a phase transition on the surface and release the dyes into the water droplets.

### 3.4. Bioanalysis

Physiological testing by commercial kits and laboratory services is becoming increasingly common. Laboratory ware such as lab-on-a-chip requires precise control of the movement of tiny droplet samples and the absence of contamination.

Li et al. demonstrated a droplet-based bioanalytical system called SLIPS laboratory to provide practical diagnostic information and rapid feedback on response to diet and drug therapy for patients with urinary tract infection [209]. Taking advantage of the low contact angle hysteresis and non-pinning contact lines of SLIPS, liquid handling procedures such as sample loading, volume measurement, droplet transport, reaction time control, and analyte detection were established. The autonomous liquid transfer allows for the processing and manipulation of physiological samples without complex sample preparation or specialized equipment.

Han et al. developed a droplet-based microfluidic system that can manipulate biofluidic droplets with low surface tension and high viscosity, oriented toward multiple bioassays and point-of-care diagnostic applications [210]. A stretchable PDMS surface with superhydrophobic and superhydrophilic patterns and a dimple structure were used to place the right amount of droplets at the right location. Single-droplet multiple colorimetric bioassays have been demonstrated as a platform for manipulating various droplets, including water, ethylene glycol, blood, dimethyl sulfoxide, and hydrogel.

Zhang et al. aimed for high-capacity transport of any biocompatible liquid free from electrical damage, thermal (high temperature) damage, and contamination from magnetic nanoparticle doping [211]. A vibration-actuated omni-droplet rectifier (VAODR) consisting of a slippery ratchet array was developed using a femtosecond laser and vibration platform. The VAODR can coordinate the interlocking between anisotropic adhesion resistance forces due to its asymmetric ratchets under the operation of periodic inertial driving forces derived from isotropic mechanical horizontal oscillations. Capable of transporting droplets of up to 2 mL and compatible with a variety of liquids, including biological fluids, medical fluids, cell culture media, alcohol solutions, and liquid metals, the VAODR is expected to enable applications in biomedical detection such as ABO blood typing and anticancer drug screening.

### 3.5. Electricity generation

Manipulating liquids is an eco-friendly power generation technology. By utilizing the electrostatic charge of a droplet, an alternating current can be formed from the electrical movement when it contacts a solid interface.

Xu et al. developed a droplet-based electric generator, which uses water droplets [212]. When the impact of a falling water droplet spreads across a non-wetting PTFE surface, it bridges the PTFE/indium tin oxide and aluminum electrodes to form an electrical system; a single droplet released from a height of 15.0 cm could power 100 commercial LEDs when it contacts the surface of that system.

Another study by the same authors also showed that the self-propelled motion of a droplet driven by a wettability gradient could lead to power generation; they demonstrated that a 25-µL self-propelled droplet could generate a peak current of 93.5 nA and maximum output power of 2.4 nW [213]. Energy conversion was proportional to the effective charge transfer at the solid – liquid interface and the separation rate, suggesting that efficient droplet movement control leads to higher energy harvesting.

### 3.6. Heat transfer

Temperature transport via droplets has applications in thermal management. For example, controlling the transport of tiny water droplets generated during the condensation process of a liquid enables improving the efficiency of a heat exchanger. If water remains on the cooling tubes of a heat exchanger, the heat transfer efficiency will be reduced (water filmwise condensation), and the water should be removed from the cooling tubes [214]. To remove water from the cooling tubes, ideally, the water should jump in the form of tiny droplets similar to mist (jumping droplet condensation) [215].

To transport micro-droplets, a structure that spontaneously transports droplets is preferred. For example, Miljkovic et al [214]. formed hydrophobic nano-needle copper oxide surfaces; the curvature gradient structure shown in Figure 8(g) promotes microdroplet coalescence and causes jumping droplet condensation. Water droplets have a higher heat transfer coefficient than air, which enables the cooling of hot materials. However, the Leidenfrost phenomenon negatively affects the use of liquids for cooling extremely high-temperature surfaces [216]. For example, when a water droplet is dropped on a solid surface (200–450°C - Leidenfrost temperature), a vapor film is formed between the droplet and the solid, preventing direct contact between the droplet and the solid. As a result, the thermal cooling efficiency decreases below that of the water droplets in contact. Therefore, design studies of interface geometries to increase the Leidenfrost temperature (the maximum temperature at which water droplets can move in contact with the solid surface) are being conducted [217].

Jiang et al. have designed a structured thermal armor (STA) structure [218]. STA consists of an array of thick pillars acting as thermal bridges and holding an insulating superhydrophilic membrane that wicks the incoming liquid. The STA structure succeeded in raising the Leidenfrost temperature above 1000°C. Conversely, Bourrianne et al. showed that the Leidenfrost temperature of water droplets could be lowered (150 °C) on long water-repellent surfaces [219].

These studies are essential fundamental studies in heat transport through droplets.

### 3.7. Water harvesting

Water is a liquid that is difficult to transport globally due to the high transportation cost. However, several regions suffer from water scarcity (particularly in arid regions), and technologies to collect water vapor (fog) from the atmosphere are considered promising. Since water vapor is minute, droplet transport techniques are used to obtain sufficient amounts of water efficiently. In nature, there are organisms, such as the Namib desert beetle, spider silk, and cactus spines, which have fog harvesting functions, and water harvesting research to date has been conducted using biomimetics originating from these organisms. Therefore, collection techniques using hydrophilic and hydrophobic wettability patterning and geometry (structure) have been developed [220].

For example, Bai et al. developed a surface with a star-shaped wettability pattern instead of employing conventional uniform patterning [221]. Integration of surface energy gradient and Laplace pressure gradient simplifies the collection of tiny water droplets and increases the efficiency of collecting water at this surface compared with surfaces with uniform wettability or circular patterns. Tian et al. demonstrated water collection by spindle-knot microfibers with cavity knots (cavity microfibers) mimicking spider silk [133]. Luo et al. have proposed a continuous system that can simultaneously achieve water collection, transport, and storage by developing a water collection platform using droplet transport with structural control and droplet condensation at low temperatures on LISs [222].

### 3.8. Desalination

Similar to fog collection, desalination of seawater is another technique that is promising for solving water resource challenges. Essential factors for desalination and water purification include increasing energy efficiency (generation of water vapor from solar energy), reducing heat loss, and controlling contamination by salt and impurities.

To achieve efficient water evaporation using solar heat, Wu et al. developed a biomimetic 3D evaporator that mimics the asymmetric capillary ratchet of a bird’s beak and utilizes the water transport phenomenon [223]. This allows for high-speed water spreading characteristics on the surface, improving water evaporation and energy efficiency. In addition, the salt crystallized locally at the top of the structure on the surface of the evaporator can be easily removed and collected, and a sustainable system is proposed.

### 3.9. Self-cleaning

The ability of a surface to self-clean, as exemplified by superhydrophobic surfaces, is one of the significant benefits of controlled wettability. Conventionally, the self-cleaning function was considered to be achieved by the droplets rolling on the surface and removing contaminants on the surface by incorporating them into the droplets.

Geyer et al. presented a mechanism for self-cleaning via confocal microscopy to observe the removal process of micrometer-order particles scattered on a surface [224]. When the droplets were moved over the contaminated surface, individual particles were picked up by advancing contact lines. When the droplet was moved further, these particles accumulated at the air – water interface at the bottom of the droplet. Because the particles lifted as the contact line receded, the frictional force was determined by the adhesion of the individual particles to the surface. The in-situ observations showed that when there were few contaminating particles, only a slight tilt of the surface was sufficient to achieve a self-cleaning function; conversely, when there were several contaminating particles, the droplets were covered with particles and became marbled, requiring additional droplets for decontamination.

### 3.10. Antifogging

Antifogging is performed by treating surfaces to superhydrophilicity with a water film in which water droplets are wetted and spread. Here, we present an example where antifogging is achieved by controlling the movement of water droplets. There are two strategies: the first method is the promotion of the coalescence of droplets on a hydrophobic surface. The energy generated during the coalescence removes the droplets from the surface (coalescence-induced jumping). The second method is the construction of hydrophilic and hydrophobic Janus surfaces and the transportation of water adhering to the surface by wettability gradients.

Zhang et al. developed a micro/nanostructured composite film composed of hollow microspheres and ZnO nanorods exhibiting superhydrophobicity even under low-temperature and high-humidity conditions [225]. They demonstrated an antifogging surface based on coalescence-induced jumping. Microdroplets accumulated and grew on the surface and gradually coalesced to form new larger droplets. The growing droplets coalesced with the neighboring microdroplets to form new large microdroplets on the surface. In this repeated coalescence and growth, the droplets were able to jump and roll off the surface due to the release of energy from the coalescence. Some of the microdroplets on the surface were removed due to the bouncing and rolling of droplets, which eventually returned the surface to a non-wetted state.

Inspired by the control of water droplet transport in the prickly pear cactus, Luo et al. developed wood slices with asymmetric wettability on the front and back surfaces connected by channels with a wettability gradient between the surfaces [226]. When atomized microdroplets adhered to the hydrophobic surface, the droplets moved to the hydrophilic side by capillary force, wetted and spread on the hydrophilic surface, and subsequently fell due to gravity. Furthermore, when the microdroplets adhered to the hydrophilic side, they did not move to the hydrophobic side but formed a water film on that hydrophilic surface and fell. Different antifogging effects were obtained on both hydrophilic and hydrophobic surfaces. Particularly on the hydrophobic surface, the transport of micro water droplets to the hydrophilic side resulted in sustained antifogging performance.

## 4. Outlook

Previous studies have discussed various strategies to control droplet motion and their critical applications. However, the following considerations should be explored for further development of the droplet motion control.

### 4.1. Enhancement of energy conversion

The minimization of the energy required to drive droplets is important. In this regard, exploration of energy conversion systems, development of the materials’ interface, minimizing the energy dissipation in manipulating systems, and discovery of new input energy sources are required. Moreover, systematic, intelligent use of sustainable energy sources like solar, electrostatic charging, wind, evaporation energy, gravity, and capillary forces, is promising.

### 4.2. Fluorine-free materials

The design of fluorine-free surfaces has been proposed considering the environmental concerns [227]. However, several studies on droplet motion control, especially in Strategy I, relied on fluorine-modified solid interfaces, including superoleophobic interfaces, self-assembled monolayers, polymer film, and slippery surfaces. Alternative fluorine-free designs with non-sticky characteristics that can replace fluorine-based engineered materials should be explored.

### 4.3. Variation of target droplets

Droplet transportation technology is not limited to simple liquids. It extends to functional liquids (including liquid metals, liquid carbon, and liquid crystals), gaseous fluids under liquid conditions, complex liquids, surfactants, and cells [140,228–230]. Additionally, by sticking or dispersing other substances as droplets, the technology can be extended to transportation, deposition, and arrangement of tiny particles of solids/gases/other complex material and, eventually, transportation of even droplet latent heat and surface charge.

### 4.4. Variation in transportation complexity

The flexibility in droplet transportation can be extended. For example, it is challenging to regulate droplet motion in three dimensions, transport nano/micrometer-sized droplets, and drive droplets in a selective, collective, or synchronized manner such as that in living species [97,231,232].

### 4.5. Applications

The research on droplet motion control is in the early stage and considers simple motion control. The research has been limited to proof-of-concept studies. Integrating droplet motion controlling system into practical devices for energy/environment, biomedical, and space applications is promising and requires further research.

## 5. Summary

This review summarizes droplet manipulation strategies based on wettability control from the perspective of mechanics. We categorized the strategies used in previous works mainly into three approaches: Those involving (i) Application of driving force to a droplet on non-sticking surfaces, (ii) Formation of gradient surface chemistry/structure, and (iii) Formation of anisotropic surface chemistry/structure. We can utilize the liquid droplet motion for various potential applications by understanding the mechanics. These mechanics are based on various parameters that must be considered, such as, the energy transfer ratio of the droplet transportation system, droplet velocity, volume, distance, cost, process, and long-term stability. Moreover, the energy conversion from the external stimuli to drive the droplet should be explored further. The discovery of new potential applications of droplet transportation is expected in various fields. We believe that this review will enable the design of droplet transportation materials/systems.
